# Editorial: Role of microbiome in diseases diagnostics and therapeutics

**DOI:** 10.3389/fcimb.2022.1025837

**Published:** 2022-09-12

**Authors:** Nar Singh Chauhan, Mitali Mukerji, Shashank Gupta

**Affiliations:** ^1^ Department of Biochemistry, Maharshi Dayanand University, Rohtak, Haryana, India; ^2^ Department of Bioscience and Bio engineering, Indian Institute of Technology, Jodhpur, India; ^3^ Faculty of Chemistry, Biotechnology and Food Science, Norwegian University of Life Sciences, As, Norway

**Keywords:** human microbiota, microbiome therapeutics, microbial diagnosis, microbiome dysbiosis, microbiota

The global microbial therapeutics product market size valued at USD 15.94 billion in 2021, is estimated to surpass 30.55 billion USD by 2030 end (https://www.futuremarketinsights.com/reports/microbial-therapeutic-products-market). This reflects the mounting interest of all disciplines besides the medical professionals for accurate and replicable microbiota profiling in diagnostic and therapeutic applications. This area has witnessed a rapid growth mainly due to huge technological advancements as well as our capabilities in understanding microbiota structure, function, their interactions with host in health and diseases (Yadav et al., 2018; Yadav and Chauhan, 2021; Yadav and Chauhan, 2022). Next generation diagnostic solutions based on microbial and microbe-based diagnostic instruments as well as microbial therapeutics promises to be a mainstay in future. (Sorbara and Pamer, 2022; Yadav and Chauhan, 2022). For example, microbial therapies that use whole microbiota, an individual microbial member, or microbial metabolites are being exploited to overcome various human illnesses (Descamps et al., 2019; Sorbara and Pamer, 2022). Microbes are pervasive in the environment because of their ability to grow and thrive in a variety of substrates (Yadav et al., 2018). Their growth and proportions could thus reflect the physiological dynamics in spatio-temporal dimensions as well as health and dysbiosis conditions. This issue deals with advancements in this new research frontier and through 24 research articles showcases the experimental studies, novel technological breakthroughs, and case studies that are being carried out to realize the concept of microbiome diagnostics and therapeutic interventions.

Mousa et al. comprehended the latest information about the human microbiota composition and physiological role in host health. Though the microbiota stabilises by adulthood, there are visible differences amongst the gut microbiome of elderly people versus younger adults (Sepp et al.). Microbiota composition could be altered by shifts in dietary patterns, medications or different lifestyle factors as well as other selection pressures such as pathogens (Maurya et al.), xenobiotics (Huang et al., Xue et al.). The shifts in microbiota composition could reflect the cause or consequence. On these lines, Saxena et al. demonstrated the influence of tobacco on oral microbiota composition and their potential role in the onset of oral cancer and Fang et al. unveiled the involvement of cervical microbiota and in the onset of HPV infections. Microbiota dysbiosis is also reported in different cancerous conditions for example squamous cell carcinoma (Jiang et al.), Benign Prostatic Hyperplasia, and Prostate Cancer (Sarkar et al.) demonstrating the utility of assessing the composition in their diagnosis. Onset as well as progression of multiple diseases, such as diabetes (Ismail et al., Du et. al.), obesity (Yan et al.), cancer (Huang et al.,Kabwe et al.), ulcerative colitis (Mirsepasi-Lauridsen et al.), tuberculosis (Wang et al.), and autism spectrum disorder (Taniya et al.) have been associated with shifts of microbial compositions. Similar perturbations were linked to different phenotypic states during progression of chronic airways disease (Chen et al.). Microbiota dysbiosis was not only observed during physiological disorders and infections but also the onset of metabolic disorders like diabetes (Ismail et al., Du et. al.) and obesity (Yan et al.). Gut microbiota could have a potential in diagnostics could be Immunoglobulin A Nephropathy (IgN) (Han et al.) as well as for monitoring bone health (Xue et al.) Perturbations in mouse models are also providing leads for the host genetics involvement in microbiota linked pathogenesis. For egusing iNOS knockout mice Aggarwal et al. demonstrated their role in diabetes progression due to gut microbiota perturbations. In another interesting study a trimethylamine oxide-induced mice model revealed the association of gut microbiota with frailty (Chen et al.). Many pharmaceutical drugs have been found to alter the composition of the gut microbiota, which is thought to be one of the mechanisms by which they exert their therapeutic effects.

The gut microbiota may be a new target for treating cardiotoxicity and cardiovascular diseases (Huang et al.).Gut microbiota could also predict responsiveness to therapy as demonstrated by Zhu et al. for monitoring erythropoietin in hemodialysis patients with anemia. Additionally, bacteriophage and selective antibiotics could be employed to engineer the gut microbiota for health benefits. Wen et al. successfully showcased the potential of fecal microbiota transplantation to treat pneumonia by improving the gut microenvironment. Another article comprehended the information of bacteriocins producing probiotics for inhibiting pathogens, modulation of the immune system, and several health-promoting functions (Anjana and Tiwari).

These research efforts highlight the enormous possibilities in microbiome diagnostics and therapeutics. However, there still remains a gap in understanding the genetic basis of host-microbiota interactions that govern inter-individual variability (Sorbara and Pamer, 2022; Yadav and Chauhan, 2022). Research that utilises phagosomes as well as metabolite-based therapeutics could be used to unravel functional aspects of disease and health conditions for translation. New sensors for microbiota metabolites are required to assess microbial activity, initiation of inflammatory conditions, and disease prognosis. Compared to other fields, microbiome therapeutics research is still in its infancy stage and comprehensive investigations are required to translate the concept of microbiome therapeutics as well as its application in precision and personalized medicine.

## Author contributions

NC and SG wrote the paper text. MM edited the text. All authors contributed to the article and approved the submitted version.

## Acknowledgments

We thank all the authors and reviewers of the paper published in this Research Topic for their valuable contributions.

## Conflict of interest

The authors declare that the research was conducted in the absence of any commercial or financial relationships that could be construed as a potential conflict of interest.

## Publisher’s note

All claims expressed in this article are solely those of the authors and do not necessarily represent those of their affiliated organizations, or those of the publisher, the editors and the reviewers. Any product that may be evaluated in this article, or claim that may be made by its manufacturer, is not guaranteed or endorsed by the publisher.
